# High Willingness to Use HIV Pre-Exposure Prophylaxis Among Transgender Women in Argentina

**DOI:** 10.1089/trgh.2016.0033

**Published:** 2016-12-01

**Authors:** Virginia Zalazar, Inés Arístegui, Thomas Kerr, Brandon D.L. Marshall, Marcela Romero, Omar Sued, M. Eugenia Socías

**Affiliations:** ^1^ Fundación Huésped, Buenos Aires, Argentina.; ^2^ Universidad de Palermo, Buenos Aires, Argentina.; ^3^ Department of Medicine, University of British Columbia, Vancouver, BC, Canada.; ^4^ British Columbia Centre for Excellence in HIV/AIDS, St. Paul's Hospital, Vancouver, BC, Canada.; ^5^ Department of Epidemiology, Brown University School of Public Health, Providence, Rhode Island.; ^6^ Asociación de Travestis, Transexuales y Transgéneros de Argentina (A.T.T.T.A.), Buenos Aires, Argentina.

**Keywords:** discrimination, HIV/AIDS, pre-exposure prophylaxis, transgender persons

## Abstract

**Purpose:** In Argentina, transgender women face a disproportionately high prevalence of HIV infection (34%). Although not currently approved in Argentina, pre-exposure prophylaxis (PrEP) may offer a potential effective HIV prevention tool for this population. In this study, we assessed the willingness to use PrEP among transgender women in Argentina.

**Methods:** Data were drawn from a nationwide cross-sectional survey conducted among transgender women in 2013. Using multivariable logistic regression, we assessed the prevalence of and factors associated with willingness to use PrEP among transgender women with negative or unknown HIV status.

**Results:** This study included 337 transgender women (278 HIV negative and 59 with unknown HIV status), most of whom had a history of sex work involvement (81.8%). Overall, 301 (89.3%) expressed willingness to use PrEP. In a multivariable analysis, having casual sexual partners was positively associated with willingness to use PrEP (adjusted odds ratio [AOR]=4.26, 95% confidence interval [CI] 1.73–10.51), while discrimination by healthcare workers was negatively associated (AOR=0.33, 95% CI 0.12–0.88).

**Conclusion:** We found high levels of willingness to use PrEP among transgender women in Argentina, suggesting that there is high perception of HIV risk in this population. However, discrimination by healthcare workers was a strong negative correlate of willingness to use PrEP, suggesting that multilevel interventions that address gender-based stigma in healthcare settings will be critical for the success of PrEP as an HIV prevention strategy in this population.

## Introduction

Globally, transgender women continued to be disproportionately represented in the HIV epidemic, with an estimated overall prevalence of HIV infection of 19.1%.^1^ In Argentina, the burden of HIV infection among transgender women is also high. Estimated HIV prevalence and incidence rates are 34% (compared with 0.4% in the general population) and 11 per 100 person-years, respectively.^2,3^ Widespread discrimination^4–6^ combined with high levels of behavioral risk factors, such as condomless sexual exposure, sex work, substance use,^7–9^ and other psychosocial and structural vulnerabilities, creates multiple barriers to healthcare access that contribute to transgender women's disproportionate burden of HIV infection as well as other health problems.^10,11^

Pre-exposure prophylaxis (PrEP) may offer a potential effective HIV prevention tool for transgender women in this multilevel risk factor context.^12^ This biomedical prevention method has been proven to reduce the risk of HIV acquisition among several groups at high risk of HIV infection, including men who have sex with men (MSM), serodiscordant heterosexual couples, and people who inject drugs (PWID).^13–19^ In these studies, PrEP efficacy estimates ranged from 44% to 86% depending on differing levels of adherence among studies. Importantly, recent trials have observed higher levels of adherence, which may reflect growing awareness of the efficacy of PrEP among participants, which in turn have resulted in higher effectiveness rates of over 90% when appropriately used.^15,20,21^

Unfortunately, participation of transgender women in PrEP trials has been minimal,^12^ and only one finished study (Preexposure Prophylaxis Initiative trial [iPrEx])^15^ had included transgender women within a larger sample of MSM,^22^ limiting the ability to draw concrete conclusions for this population.

In Latin-American countries, Peru and Brazil have been in the frontline of PrEP implementation research. Peru has hosted three sites in the iPrEx trial and its open-label extension (iPrEx OLE)^23^ and conducted several studies on providers' and sexual/gender minorities' expectations and attitudes regarding PrEP, as well as cost-effectiveness analysis of scaling up PrEP in their local context.^15,22,24^ Brazil has also hosted three sites in iPrEx and iPrEx OLE and currently has an ongoing PrEP demonstration project in MSM and transgender women (PrEPBrasil).^25^

Despite the World Health Organization's broad recommendation for PrEP use in individuals at risk of HIV, including transgender women,^26^ there are no specific guidelines for this population. Likewise, although PrEP is being increasingly adopted in several countries, including the United States (2015), South Africa (2015), Kenya (2016), France (2016), and Norway (2016), there is no mention of transgender women-specific recommendations in any of these countries' policies.^24^

Argentina's current HIV guidelines do not recommend PrEP based on concerns about potential risk compensation behaviors and low adherence, the high costs, and nonexistence of local studies.^27^ These concerns are also reflected by the low willingness to offer PrEP to different at-risk populations, including transgender women among Argentinean health providers,^28^ despite PrEP potential to decrease HIV incidence in this population.

Alongside health system and providers' concerns of PrEP effectiveness among transgender women, there is limited and conflicting evidence of transgender-specific factors related to acceptability of PrEP, with studies indicating a wide range of willingness to use PrEP from 37% to 96%. This wide range could be the result of differences in the measurement of PrEP acceptability in the few surveys that have focused on transgender women, without including them in the MSM category: two in Peru, one in Thailand, and another in United States.^29–33^ Other factors that may explain this wide range of acceptability are different cultural and economic factors in each of these settings (e.g., misconceptions about PrEP, potential out-of-pocket costs, sexual practices).^31^

Therefore, as there is limited knowledge regarding transgender women's acceptability of PrEP in the local context, the objective of this study was to investigate the prevalence and correlates of willingness to use PrEP among transgender women in Argentina, with a particular focus on the role that social–structural factors may play in facilitating or hindering its implementation and rollout.^12,34^

## Methods

### Study design and sample

Data for the present study were drawn from a nationwide cross-sectional survey among 498 transgender individuals in Argentina, conducted between June and December of 2013 by Fundación Huésped (a nonprofit HIV/AIDS organization) and the Association of Transvestites, Transsexuals, and Transgender of Argentina (ATTTA). Self-identified transgender individuals were recruited through extensive outreach with a focus on sex work venues and community-based organizations known to be frequented by transgender persons. To maximize representativeness of the transgender population in Argentina, snowball sampling was combined with quota sampling.^35,36^

Quotas were calculated based on data collected by the national registration office (Registro Nacional de las Personas [RENAPER]) and other reports of sociodemographic characteristics of transgender individuals in Argentina. With the purpose of obtaining a sample size of 500, quotas were set for gender identity (41 transmen, 459 transwomen); six age categories (31: 14–19 years, 211: 20–29 years, 171: 30–39 years, 66: 40–49 years, 18: 50–59 years, 3: 60+ years); five educational levels (3 no studies, 31 incomplete primary education, 107 complete primary education, 185 incomplete high school education, 174 complete high school education or greater); and the six Argentinean regions (153 Buenos Aires metropolitan area, 161 Pampeana, 70 Noroeste, 41 Nordeste, 38 Cuyo, 37 Patagonia). A more detailed description of our sample and recruitment procedures has been described previously.^5,37,38^

After providing written informed consent, eligible individuals completed a semistructured questionnaire administered by trained peer interviewers. The questionnaire collected a range of variables, including data regarding sociodemographic characteristics, gender enhancement or transition procedures, self-reported HIV status, HIV knowledge and willingness to use biomedical prevention tools (e.g., antiretroviral therapy [ART] for HIV prevention, PrEP, microbicides), interactions with police, healthcare access, housing, education, work, and experiences of stigma and discrimination in these settings.

The study was approved by the institutional ethics committee of Fundación Huésped and all participants received a $100 ARS (approximately $10 USD) reimbursement for their time and participation in the study. The current analysis was restricted to transgender women with negative or unknown HIV status.

### Primary outcome

The primary outcome of interest was willingness to use PrEP, defined as answering yes to the following question: “If the possibility of taking a daily pill to prevent HIV existed, would you be interested in taking it?”

### Explanatory variables

Based on previous studies examining willingness to use PrEP among different key populations,^29,32,39–43^ we considered a range of individual and social–structural factors that were hypothesized to influence willingness to use PrEP among transgender women in our setting. These included age (per year older); immigration status (immigrant vs. Argentinean born); place of residency (Buenos Aires metropolitan area, the biggest urban center in Argentina, vs. others); higher educational level (≥high school education vs. <high school); history of sex work involvement (yes vs. no); history of sexually transmitted infections or STIs (yes, at least one STI vs. no); stable (i.e., Do you have a stable partner—a person with whom you have frequent sexual intercourse and a committed relationship?; yes vs. no) and casual partners (i.e., Do you have casual or occasional sexual partners?; yes vs. no); and consistent condom use with different types of partners, including sex clients and casual and stable partners (defined as always vs. sometimes or never). Additionally, to be consistent with previous research examining PrEP acceptability among transgender women,^32,44–46^ we included a variable addressing lifetime gender-based discrimination in healthcare settings (yes vs. no), assessed with the question: “Due to your transgender identity, have you ever experienced discrimination from physicians, nurses, or other healthcare workers?” as defined in previous studies with this population.^5,37,38^

At the time of survey administration (2013), the effectiveness of PrEP in different populations (e.g., sex workers, MSM, transgender women) and under different real-world conditions was unclear.^13–15,19,21^ Thus, drawing on previous studies on PrEP acceptability,^31,47^ in a subanalysis, we explored willingness to use different HIV prevention methods (i.e., condoms and PrEP) alone or in combination, under a hypothetical scenario with low efficacy of PrEP (i.e., PrEP not as effective as condoms for HIV prevention).

### Statistical analyses

As a first step, Pearson's chi-square test for categorical variables and the Wilcoxon rank sum test for continuous variables were performed to examine bivariable associations between independent variables of interest and willingness to use PrEP. All variables found to be associated with the outcome at *p*<0.10 were then entered into a multivariable logistic regression model. Two-sided *p*-values as well as unadjusted and adjusted ORs (AOR) with 95% confidence intervals [CIs] are reported. All analyses were performed with SPSS version 22.0.^48^

## Results

The original study included 452 transgender women. Of these, 103 (22.8%) identified as HIV positive and 12 (2.6%) did not provide a valid response to the primary outcome and thus were excluded from the present analysis. The final analytic sample included a total of 337 (74.6%) transgender women (278 self-reported HIV negative and 59 with unknown HIV status).

Characteristics of study participants stratified by willingness to use PrEP are presented in Table 1. The median age was 29 (interquartile range [IQR]: 24–37), the majority reported a history of sex work involvement (81%) and almost half reported that have been diagnosed with at least one STI in their lifetime (41.9%). Overall, 301 (89.3%) transgender women stated that they would be willing to use PrEP if it became available. In the low-efficacy PrEP hypothetical scenario, only 9.7% of participants would still use PrEP exclusively, while 37.3% reported that they would only use condoms, and 53.4% reported that they would use both methods.

**Table 1.** **Baseline Characteristics and Bivariable Logistic Regression of Factors Associated with Willingness to Use PrEP Among Transgender Women (*n*=337)**

		Willingness to use PrEP, *n* (%)			
Characteristic	Total (*n*=337)	Yes (*n*=301)	No (*n*=36)	Odds ratio (95% CI)	*p*
Age, median (IQR)	29 (24–37)	30 (24–37)	27 (23–34)	1.02 (0.98–1.06)	0.254
Foreign born					
Yes	22 (6.5)	19 (6.3)	3 (8.3)	0.74 (0.20–2.63)	0.643
No	315 (93.5)	282 (93.7)	33 (91.7)		
Place of residency					
Buenos Aires city	176 (52.2)	151 (50.2)	25 (69.4)	2.25 (1.07–4.75)	0.029
Other	161 (47.8)	150 (49.8)	11 (30.6)		
Highest educational attainment					
High school or greater	117 (34.8)	100 (33.3)	17 (47.2)	0.55 (0.27–1.12)	0.098
Less than high school	219 (65.2)	200 (66.7)	19 (52.8)		
Discrimination by healthcare workers^a^					
Yes	225 (66.8)	195 (64.8)	30 (83.3)	0.36 (0.14–0.91)	0.026
No	112 (32.2)	106 (35.2)	6 (16.7)		
Stable partner^b^					
Yes	123 (37.5)	110 (37.5)	13 (37.1)	1.01 (0.49–2.10)	0.963
No	205 (62.5)	183 (62.5)	22 (62.9)		
Consistent condom use with stable partner^b,c^					
Yes	44 (36.1)	38 (34.1)	6 (46.2)	0.62 (0.19–1.99)	0.423
No	78 (63.9)	71 (65.1)	7 (53.8)		
Casual partners^b^					
Yes	227 (85.2)	256 (87.7)	21 (63.6)	4.06 (1.84–8.95)	<0.001
No	48 (14.8)	36 (12.3)	12 (36.4)		
Consistent condom use with casual partners^b,c^					
Yes	184 (66.9)	171 (67.1)	13 (65.0)	1.09 (0.42–2.84)	0.851
No	91 (33.1)	84 (32.9)	7 (35.0)		
Sex work^a^					
Yes	265 (81.8)	243 (83.5)	22 (66.7)	2.53 (1.52–5.56)	0.018
No	59 (18.2)	48 (16.5)	11 (33.3)		
Consistent condom use with sex clients^a,c^					
Yes	201 (76.1)	184 (76.0)	17 (77.3)	0.93 (0.33–2.64)	0.896
No	63 (23.9)	58 (24.0)	5 (33.3)		
STI^a,d^					
Yes	131 (41.9)	114 (41.0)	17 (48.6)	0.73 (0.36–1.48)	0.393
No	182 (58.1)	164 (59.0)	18 (51.4)		

^a^ Denotes lifetime experience.

^b^ Denotes current behavior or activity.

^c^ Among participants with that type of partner.

^d^ Among participants with a valid response to this question.

CI, confidence interval; PrEP, pre-exposure prophylaxis; STI, sexually transmitted infection.

The results of the bivariable analysis of factors associated with willingness to use PrEP are shown in Table 1. Residency in Buenos Aires (OR=2.25, 95% CI 1.07–4.75), history of sex work (OR=2.53, 95% CI 1.52–5.56), and having casual partners (OR=4.06, 95% CI 1.84–8.95) were positively and significantly associated with willingness to use PrEP. In contrast, previous experience of discrimination by healthcare workers was associated with reduced odds of willingness (OR=0.36, 95% CI 0.14–0.91).

In the final model (Table 2), only having casual partners (AOR=4.26, 95% CI 1.73–10.51) was positively associated with willingness to use PrEP, while discrimination by healthcare workers remained negatively associated with willingness to use PrEP (AOR=0.33, 95% CI 1.12–0.88).

**Table 2.** **Multivariable Logistic Regression Analysis of Factors Associated with Willingness to Use PrEP Among Transgender Women in Argentina**

Variable	Adjusted odds ratio	95% Confidence interval	*p*
Highest level of educational level (≥high school vs. less than high school)	0.48	(0.21–1.05)	0.069
Place of residency (Buenos Aires city vs. other)	2.07	(0.92–4.66)	0.076
Sex work^a^ (yes vs. no)	1.73	(0.71–4.23)	0.227
Discrimination by healthcare workers^a^ (yes vs. no)	0.33	(0.12–0.88)	0.027
Casual partners^b^ (yes vs. no)	4.26	(1.73–10.51)	0.002

^a^ Denotes lifetime experience.

^b^ Denotes current behavior or activity.

## Discussion

This study found high levels of willingness to use PrEP among transgender women in Argentina. Almost 90% of participants reported that they would be willing to use PrEP if it became available, a relatively higher prevalence than what has been reported in previous studies among transgender women (37%–76.6%)^29,30^ and other at-risk populations, such as MSM, serodiscordant couples, and PWID (28%–80%).^31^ However, our findings are similar to another study in Peru that found 96% prevalence of willingness to use PrEP among MSM and transgender women.^33^ Differences among studies may be due to different perceptions of HIV risk among marginalized populations and cultural settings, PrEP knowledge, and motivations to use PrEP.^31^ Further qualitative research may help in better understanding these differences that may help in tailoring prevention approaches to specific populations.

Concerns about risk compensation behaviors that could offset any potential benefit of PrEP are frequently cited as reasons to discourage PrEP implementation as a public health strategy.^27,49^ However, most studies to date, including clinical trials and demonstration projects, indicate that this concern is unfounded and, on the contrary, suggest that PrEP may even lead to reduced risk-taking behaviors.^40,45,50^

Results from our study are in line with this evidence as more than 90% of the study sample stated that if PrEP was not as effective as condoms for HIV prevention, they would consider using condoms either alone or in combination with PrEP. Although these findings should be taken with caution, they suggest that many transgender women may consider using PrEP in combination with condoms for additional protection against HIV as well as for other STIs. This is especially important to address and expand in future studies given that recent research has shown an alarming increase in STIs among MSM using PrEP,^51^ and transgender women already have a high incidence and prevalence of STIs (41.9% self-reported in the present study). The implementation of PrEP should be an opportunity to include transgender women in comprehensive prevention programs offering frequent HIV testing, STI screening, and condom promotion.

A particularly concerning finding of this study is that despite the high overall observed willingness to use PrEP, previous experiences of gender-based discrimination by healthcare workers were an independent and strong negative correlate of willingness. This is in agreement with previous studies indicating that fear of or actual experiences of discrimination in healthcare settings due to transgender identity may negatively impact transgender women's uptake of PrEP or other needed health services.^5,8,32,44,46^ Collectively, these results emphasize the importance of gender-based discrimination as a barrier to healthcare for this population. Thus, there is an urgent need to educate and train healthcare providers to improve communication and trust with transgender women to create gender- and culturally welcoming healthcare setting environments.^5,6,11,52^ This will be critical for any potential future effort of PrEP implementation. In such regard, recent implementation of progressive laws specific to transgender individuals in Argentina, such as the Gender Identity Law, provides a human rights-based framework for emerging efforts addressing the multiple health inequalities faced by transgender women.^38^

Another relevant finding of the present analysis is that participants with casual sex partners had increased odds of willingness to use PrEP. This finding alongside a reported high prevalence of consistent condom use with casual partners—higher than with stable partners or clients—suggests that there might be a high perception of risk of HIV with this type of partner, which is not mediated by strictly business aspects of sex work, nor by trust, affection, and/or commitment of stable relationships.^10^ Indeed, risk negotiation with sex partners has been shown to be an important factor for PrEP acceptability and adherence.^43,46^ Among transgender women, low self-efficacy in risk negotiation is the result of transphobia and social isolation, leading to the need for gender affirmation and love and desire by male partners, even to riskier sex with riskier partners.^9,12,46,53^ Altogether, our results highlight the need to consider prevention strategies that take into account this type of partner regardless of transgender women's involvement in sex work.

### Limitations

The present study has several limitations. First, as the survey was not specifically designed to assess willingness to use PrEP, there are many important unanswered questions that should be addressed in future studies, including risky sex behavior such as condomless anal sex or sex with HIV-positive partner, the impact on willingness to use PrEP of alternative dosing schedules, potential costs, and interactions with feminizing hormones. In addition, this high hypothetical willingness to use PrEP may not be maintained once PrEP is actually available. Second, the exclusion of participants who refused to answer questions regarding PrEP may have introduced selection bias. Given the small number of refusals, we anticipate the magnitude of this bias, if any, to be small. Third, the results may not necessarily be generalizable to all transgender women in Argentina. In particular, because much of the recruitment was conducted in sex work venues, transgender women engaging in sex work are likely to be overrepresented. However, the final sample was very similar to that of the quota recruitment goals calculated using government statistics provided by the national registration office (RENAPER). Thus, we have reasonable confidence that the data presented in this study are reflective of experiences that transgender women face across Argentina. Fourth, as the study relied on self-reported information, the number of self-reported HIV-negative participants may have been overestimated due to usually low levels of HIV status awareness among transgender women^8^ or social desirability bias. Fifth, because survey administration was in 2013, in a time when PrEP effectiveness was unclear, a question regarding willingness to use PreP under different efficacy scenarios was added. This could have influenced participants to consider responding they would use condoms alone or in combination with PrEP. Nonetheless, the high frequency of intention to use condoms alone or in combination with PrEP among transgender women is worthy of note and should be addressed in future studies. Finally, the questionnaire was administered by interviewers and thus social desirability might have led to overestimation of PrEP willingness and intention to use condoms, as well as underestimation of risky sex behavior, among others. In addition, the prevailing stigma and discrimination against people living with HIV could have led to underreporting of HIV-positive status. However, surveys were administered by trained peer interviewers, who were well-known and trusted ATTTA leaders, with gender identities and life experiences in common with participants, which may have decreased the extent of social desirability bias and might have minimized the anticipation of stigma and discrimination.

## Conclusion

In summary, we found high levels of willingness to use PrEP among transgender women in Argentina, particularly among those with casual partners, highlighting the need to consider this type of partnership for future design of studies and prevention strategies among transgender women.

Furthermore, particularly relevant from a health system point of view, gender-based discrimination by healthcare workers was a strong negative correlate of willingness. These results suggest that health policy efforts in Argentina should focus on tackling gender-based stigma and discrimination in healthcare settings as an essential step for achieving comprehensive care for transgender women. Sustained access and uptake of PrEP as part of a comprehensive prevention approach, including ART for transgender women living with HIV, will be key to reducing the burden of HIV infection among this marginalized population.

## Abbreviations Used

AOR: adjusted odds ratio
ART: Antiretroviral therapy
ATTTA: Association of Transvestites, Transsexuals, and Transgender of Argentina
CI: confidence interval
iPrEx: Preexposure Prophylaxis Initiative trial
MSM: men who have sex with men
OLE: open-label extension
PrEP: pre-exposure prophylaxis
STI: sexually transmitted infection
